# DMPA-SC self-injection experiences of clients and providers in Uganda: the role of community health workers in reproductive self-care service delivery

**DOI:** 10.1186/s12905-025-03850-9

**Published:** 2025-07-10

**Authors:** Jane Cover, Allen Namagembe, Barbara Kunihira, Cecilia Nantume, Andrew Secor, Fiona Walugembe

**Affiliations:** 1 PATH, Seattle, WA USA; 2 PATH, Kampala, Uganda

**Keywords:** Self-care, Contraceptive self-care, Self-injection, DMPA-SC, Community health worker, Uganda

## Abstract

Accelerating task sharing for family planning and contraceptive self-care can minimize the impact of a projected shortage of 18 million health care workers by 2030. This cross-sectional study assessed the potential of community health workers (CHWs) to offer family planning counseling and self-injection training comparable in quality to that provided by clinic health workers. The study employed exit interviews with 240 injectable clients and 80 of the providers who conducted their counseling, from 43 purposively selected public sector clinics across eight districts of Uganda. The study examined the feasibility, acceptability, and quality of contraceptive service delivery, including self-injection training for women interested in self-care. The study also measured awareness and interest in self-injection among women and assessed provider receptivity to offering self-injection. Data was analyzed using STATA 14.2, using chi square and t-tests to measure for any bivariate significant differences at conventional significance levels for two-sided tests (*p* < .05). Results from interviews with injectable clients revealed a high level of interest in self-injection, ranging from 48 to 80% depending on the metric used. With respect to the quality of family planning counseling, interviews with clients indicate that CHWs provide a higher quality of care than that offered by their clinic-based counterparts, whether measured by the method information index plus (MII +) or the Quality of Contraceptive Counseling (QCC) scale. In terms of self-injection training, CHWs were significantly more likely to conduct individual training, ensure private time with the client (if trained in a group), show the client a job aid, and advise on disposal. In terms of receptivity to self-injection, both groups of providers expressed favorable views in general, though a substantial share would place restrictions on who can self-inject, limiting access for covert users (41%), adolescents (49%), and new users (58%). CHWs self-reported as better able to accommodate the time required for high quality family planning counseling and self-injection training. The findings from this study should reassure stakeholders that, when provided with appropriate, competency-based training and supervision, CHWs can help to fill a looming human resource shortfall, reinforcing family planning service delivery while reaching women who face profound geographic access challenges.

## Background

Contraceptive self-care offers women the promise of enhanced privacy, reduced health inequities, and greater self-determination and autonomy over contraceptive decision making and fertility management. While practiced for millennia, the concept of self-care has generated enormous interest since the publication of the World Health Organization’s (WHO) self-care guidelines in 2019, updated in 2022 [1]. Among WHO’s recommended self-care interventions, contraceptive self-injection with subcutaneous depot medroxyprogesterone acetate (DMPA-SC), which was first introduced outside of a research setting in 2018 in Uganda, is currently being offered in more than 30 countries [2]. However, only around one in five client visits for DMPA-SC involves self-injection (SI) rather than provider administration (among 15 countries where progress with SI scale up is closely monitored) [2]. Performance Monitoring for Action (PMA) surveys conducted in Nigeria, Democratic Republic of Congo (DRC), Burkina Faso, and Kenya showed low levels of SI awareness, use, and intent to use as of winter, 2020–2021 [3]. (The PMA project conducts frequent population-based cross sectional and longitudinal surveys to collect actionable family planning data across nine countries and recently added questions about self-injection to their instruments in select countries. Readers can learn more about the PMA from their website: www.pma.org.) In light of these patterns, some have speculated that some donors, implementing partners, and ministry of health stakeholders may have overestimated women’s interest in self-administration of injectable contraception [3]. While certainly possible, it may also be the case that bringing this innovative contraceptive intervention to scale is more complicated than anticipated.

In most countries, there is no direct-to-consumer channel for women to learn self-injection; providers serve as gatekeepers, deciding when, and to whom, they offer SI training. Numerous studies have shown that, when well-trained, women are readily able to manage SI, including women who have not attended school [4–7]. However, it takes time, on average ~ 30 min, to train a woman well-enough that she feels confident to inject herself [8]. In routine (non-research) settings, many women initially trained to self-inject decline to do so at the end of their training and the quality of SI training offered to clients impacts their willingness to self-inject [8]. Perhaps not surprising given global human resource constraints in the health care sector, studies show that a substantial share of clinic providers report that it is challenging to find time to offer SI training [9]. If providers find that their training is often unsuccessful, with clients declining to self-inject at the end of training, they may become disincentivized to offer the training, concluding that women in their community are not capable of learning self-injection.

Out of concern for costs, complexity, and the capacity and competence of lay health workers, many countries have opted to offer SI service delivery only via clinic providers. In some settings, the role of CHWs begins and ends with educating women that there is a self-injection option and referral to clinics, or CHWs may be authorized to administer DMPA-SC injections and resupply women but are not permitted to train women for SI. In short, access to SI services is limited to clinic settings in many countries. Among 18 countries scaling SI, 13 have authorized SI services (injection administration, SI training, as well as resupply) through CHWs; however, as of this writing, just three – Uganda, Malawi, and Nigeria—have implemented the policy by training a substantial share of CHWs in how to counsel women for SI [2].

This relatively low degree of involvement of CHWs in SI service delivery is somewhat surprising given global recommendations around task sharing for injectables. After an extensive review of the evidence, the WHO issued recommendations that community health workers can safely and effectively task share family planning, including administration of injectable contraception (2012 and 2017) [10]. USAID identified task sharing for family planning service delivery with CHWs as a proven high impact practice (HIP), noting that CHW programs are a cost-effective approach to increase contraceptive use, while reducing inequities in access to health care experienced by women with less education and fewer resources, and/or who live in more rural areas [11] The HIP brief specifically mentions injectables among the methods that CHWs can safely administer. More recently, a systematic review of evidence focused on CHWs in sub-Saharan Africa found that CHWs competently and safely administer injectable contraception, with “equivalent or higher performance” relative to clinic providers [12]. With respect to training women for SI specifically, a randomized control trial found higher continuation rates for self-injecting women (relative to women who received the injection from a provider), many of whom were served by CHWs; however, the study was not designed to measure differences in continuation between SI clients served by CHWs and those served by clinic providers [4]. More recently, a study evaluating SI service delivery in Uganda found comparable injection proficiency among women trained to self-inject by CHWs, relative to women trained by clinic providers, despite lower average levels of education among the clients of CHWs [8].

CHWs have long been integral to family planning service delivery in Uganda and have played an important role in scaling up self-injection. The Village Health Teams (VHT) program in Uganda, established in 2001, comprises over 150,000 community health workers selected from local communities to provide basic healthcare services and education at the household level. There are no specific educational requirements, though VHTs are expected to be literate in a local language. VHTs are remunerated with a monthly stipend of 10,000 Uganda shillings ($2.5 USD) for transport, an amount that is insufficient to cover out-of-pocket costs, contributing to low motivation and morale. Training, supervision, and provision of equipment and supplies are provided primarily by the District Health Team, often with financial support from implementing partner organizations. Despite challenges, VHTs contribute significantly to community health, offering referrals, health education, and primary healthcare services, including reproductive, maternal, newborn, and child health (RMNACH) services, distribution of treatments for integrated community case management (ICCM), HIV, malaria, tuberculosis, and other conditions. Notably, VHTs have been offering DMPA-SC since 2013 and over 15,000 VHTs had been trained as of December 2023 to offer DMPA-SC self-injection services in their communities, highlighting the program’s commitment to expanding access to family planning services. A description of Uganda’s approach to introduction and scale up of self-injection is described elsewhere [13].

Because of its integration of CHWs into the family planning program, and SI service delivery specifically, the Ugandan country context presents an opportunity to better understand the potential of the community service delivery channel. This study explores several key questions related to how and in what ways self-injection services are delivered, as well as questions of client demand for SI. Specifically, we address the following key questions:

1. To what extent are women who seek family planning from CHWs interested in SI, given the convenience of CHWs present in nearby communities?
2. From the provider perspective, how receptive are different cadres to offering SI services?
3. What feasibility challenges do providers face when adding SI training to their family planning offerings?
4. What is the level of quality of family planning counseling and SI training as reported by clients receiving services at clinics and those receiving services in the community?
5. Fundamentally, despite lower levels of training and education, are CHWs able to offer family planning and SI services of comparable quality to those offered by clinic providers?

## Methods

The study was a cross-sectional evaluation of the SI program in eight districts in Uganda, with data obtained through structured interviews with family planning clients and providers. The field team was comprised of five female research assistants supervised by a study coordinator, all of whom were trained in research ethics and the informed consent process, interviewing, and data management.

### Sampling, recruitment, and eligibility

The sampling strategy for participating facilities (and their catchment areas) was purposive, identifying from routine HMIS data a mix of low, medium, and high-volume sites, defined as such by the percent of DMPA-SC visits that were for self-injection. Cut points were derived based on the median and interquartile range (for percent SI) for the full distribution of facilities. From among the 137 districts in Uganda, not all of which have comprehensively rolled out provider training, sites were drawn from districts where training in how to counsel women for SI was considered to be complete, as of December 2021. For each of eight selected districts (Alebtong, Buikwe, Gulu, Jinja, Kassanda, Mayuge, Nakasangola and Oyam), up to two facilities of each type – low, medium, and high SI percentage based on the full distribution – were identified to capture a wide variety of experiences with the SI program. Facilities with zero DMPA-SC visits were eliminated from the sample under the assumption that the facility was likely out of stock or did not have any providers trained to offer SI (due to facility transfer, retirement, etc.) A total of 43 facilities were drawn into the sample (not all districts had two sites with low, medium, and high SI volume).

Six clients were recruited from each facility/facility catchment area, including three through exit interviews at the clinic and three randomly drawn from lists of injectable clients seen by CHWs. Clients were eligible to participate in the study if they were at least 18 years of age, able to understand and speak the local language, and had received injectable contraception, either on the day of the clinic visit, or within the past two weeks if a client of a CHW. Clients were recruited at the end of their family planning visit in-person at the clinic, selecting at least one self-injecting client, if available. To identify community clients, the CHWs were given recruitment scripts to screen for interest in the study from clients they had served in the past two weeks. The names of clients, mode of injectable delivery, and date of service for each client interested in learning more about the study were forwarded to the evaluation team. The evaluation team employed stratified random sampling to select three clients to recruit (at least one of which would be a self-injecting client, if available).

Providers were recruited after all client interviews were completed for a given facility and catchment area to reduce the influence of the Hawthorne Effect (providers changing their counseling practices with knowledge of study purpose). For each location, two providers were recruited – one based at the facility and one from the community. Providers were at least 18 years of age and offer family planning services at the selected facility or surrounding community. Specifically, in the clinic setting, the provider who was offering family planning services on the day clients were interviewed was selected, preferencing one with a client who was interviewed (if more than one family planning provider). For community providers, the facility in-charge provided the names of three CHWs attached to the facility who offer family planning services. (These same three CHWs provided the client lists used to select clients, as described above). If the clients interviewed were served by different CHWs, simple randomization was used to select one to interview.

### Data collection, key measures, and analysis

Research assistants conducted one-on-one, in-person structured survey interviews after obtaining informed consent, in a location with auditory privacy, and in the preferred language of the participant. Data was collected electronically using ODK and uploaded daily to KoboCollect.

We included several measures in the client instrument to better understand the content and quality of information provided during family planning counseling. The Method Information Index Plus (MII +) is a measure frequently used in surveys to assess whether women receive adequate information about family planning during counseling [14]. The MII + is comprised of four questions: 1) Whether additional methods than the one chosen were discussed; 2) Whether the provider discussed side effects for the method chosen; 3) Whether the client was given information about what to do in the event of problems; and 4) Whether the provider mentioned the possibility of switching methods if not satisfied. The index is expressed as the percent of women who respond affirmatively to all four questions.

To complement the MII +, we included two additional items to assess the nature and quality of the information exchange, specifically, whether the client was asked if she had any questions and the extent to which the information provided to her was clear.

While the MII + is easy to use and simple to measure via surveys, it focuses uniquely on what information is provided. Other measures have attempted to generate a more nuanced understanding of counseling quality by encompassing dimensions beyond information. The Quality of Contraceptive Counseling Scale (QCCS) is a 22 item question series, validated in multiple country settings, that reflects the quality of information exchanged (10 items), interpersonal relationships (7 items) and disrespect/abuse (5 items) [15]. Responses are measured using a Likert scale, and coded such that a higher average score reflects a more positive assessment of the counseling session (negatively expressed items were reverse coded before calculating the score), for each dimension as well as for the total QCCS.

To better understand the level of client awareness of SI services, we included the question used in select PMA surveys, “Have you heard of self-injectable contraception?”. To understand client interest in SI, we included the PMA measure, “Would you consider using self-injection in the future?”, which is asked in the PMA surveys of non-users who indicate intent to use injectable contraception in the future. For the present study, the question was asked of all women not currently self-injecting. We supplemented these two measures with one that distinguishes with greater nuance the level of interest in SI. Specifically, our measure was intended to reduce the influence of social desirability, or the tendency of participants to give favorable responses in surveys and is worded as follows: “We want to understand how interested personally you are in self-injection with Sayana Press (DMPA-SC). There is no correct answer. We want to know your honest feelings. Which sentence best describes you?”, with response options of, 1) “I currently self-inject or have self-injected in the past”; 2) “I am very interested in trying self-injection”; 3) “I am somewhat interested in self-injection and might consider it”; 4) “Self-injection is just not for me.”

With respect to self-injection training experiences, we included questions to understand the nature and quality of self-injection training (in the client instrument), including questions that reflect best practices in self-injection training, such as using a job aid, demonstrating the injection, focusing on the four critical steps, and supervising and coaching during the injection (among others).

With respect to the provider instrument, the survey captured data on background characteristics, working conditions and staffing, their own training experience, as well as their experience with and approach to training clients to self-inject, the degree of confidence they feel in their ability to train clients to self-inject, and perspectives on self-injection.

To understand receptivity of providers to self-injection, we included a series of questions to identify general reservations about self-injection, the nature of any restrictions that providers impose on who they will train in self-injection, and overall self-injection receptivity. Responses to the questions on receptivity to self-injection are measured using a Likert scale, with negatively expressed items reverse coded before calculating a total numeric score.

Given access challenges that adolescents often face when seeking family planning services generally and hormonal contraception specifically [16–19], the provider instrument included several questions to better understand provider receptivity to serving adolescent clients, in general, as well as restrictions on adolescent use of injectable contraception specifically.

The surveys were not pretested since most measures had been validated previously either in Uganda specifically, or in another Sub-Saharan African country. To ensure comprehension, responses were closely monitored and ambiguities in understanding promptly addressed during regular debriefings with the team. Because the surveys were electronic, additional response categories were added or question wording corrected, as needed, during field work.

Data was analyzed using STATA 14.2, using chi square and t-tests to measure for any significant differences at conventional significance levels for two-sided tests (*p* < .05). The study was not powered to test any specific hypothesis. Our analyses focused on differences in the experience of clients visiting CHWs and those visiting clinic providers, as well as differences between clinic- and community-based providers’ perspectives and practices related to family planning counseling, and self-injection specifically.

### Ethical considerations

All participants provided written informed consent. Ethical approval was granted by Ugandan National Health Laboratory Services Research and Ethics Committee (UNHLSREC) and the Ugandan National Council for Science and Technology (UNCST).

## Results

A total of 240 clients were recruited, evenly split between clients served by CHWs and those served by clinics. A total of 80 providers, 40 clinic-based and 40 community based, were enrolled.

### Provider characteristics

The profile of CHWs differs significantly from that of clinic providers, beyond the expected educational and job cadre differences. CHWs are significantly older, with more years of experience in family planning service delivery, and more likely to be male than the clinic-based health workers (Table 1).

**Table 1:** Characteristics of providers surveyed

	**CHW (*n* = 40)**	**Facility HW (*n* = 40)**	***P* value**
Age, mean (SD)	***46.6 (1.7)***	***33.8* (1.2)**	.000
Sex, (female) n (%)	***21 (52.5%)***	***38 (95.0%)***	.000
Cadre, n (%)			.000
Midwife/Nurse	***0***	***31 (77.5%)***	
Nursing assistant	***0***	***4 (10.0%)***	
CHW	***40 (100%)***	***4 (10.0%)***	
Other	***0***	***1 (2.5%)***	
Education, n (%)			.000
Primary	***11 (27.5%)***	***0 (0%)***	
Secondary O	***21 (52.5%)***	***4 (10.0%)***	
Post secondary Vocational	***5 (12.5%)***	***19 (47.5%)***	
Secondary A/Post A Vocational	***2 (5.0%)***	***14 (35.0%)***	
University	***1 (2.5%)***	***3 (7.5%)***	
Months providing FP services at facility/in community (SD), mean (SD)	***107 (12.3)***	***46.4 (6.4)***	.000

Bolded, italicized results are significant at *p* <.05

### Provider training, supply constraints and delivery of SI services

With respect to training for providers, sizeable minorities of providers had not been trained in how to counsel women for SI, including 40% of clinic providers (Table 2). Similarly, many had not received any post-training supportive supervision that included discussion of SI, with CHWs significantly less likely to have received a supportive supervision visit. Around one third of providers indicated difficulty maintaining sufficient supplies of DMPA-SC to meet demand. With respect to their experience offering SI services, about three quarters of providers of both types reported that they had conducted SI training within the previous month. For the relatively small number who had not conducted training, three barriers rise to the top for CHWs – lack of stock, lack of training for the provider in how to counsel women for SI, and the perception that women are not interested in SI. Clinic providers also identified lack of stock and lack of training, while also noting lack of time to train women.

**Table 2:** SI training experience and identified challenges with SI service delivery

	**CHW (*n* = 40)**	**Facility HW (*n* = 40)**	***P* value**
Provider received training in DMPA-SC SI, n (%)	29 (72.5%)	24 (60.0%)	.237
Provider received post-training supportive supervision, n (%)	***17 (58.6%)***	***20 (83.3%)***	.050
Supply constraints for DMPA-SC, last six months, n (%)	10 (31.3%)	14 (35.0%)	.737
Conducted SI training for clients last 30 days, n (%)	31 (77.5%)	30 (75.0%)	.793
Reasons for not training women, n	*N* = 9	*N* = 10	NA
Provider not trained	4	3	
Lack of stock	3	4	
Lack of time	0	3	
Lack of materials (job aids)	1	1	
Women are not interested	3	2	
Away from site	0	1	
Number trained last 30 days, mean (SD)	12.4 (2.3)	15.5 (2.6)	.364
Self-injection among those trained (estimated), %	44.7%	49.7%	.593
SI training difficulty (‘Very easy’), n (%)	11 (30.6%)	11 (31.4%)	.937
Confidence in ability to train women (‘Very confident’), n (%)	26 (89.7%)	18 (75.0%)	.157
Time in minutes required for training, mean (SD)	34.2 (5.0)	23.5 (3.0)	.072
Feasibility of training time required (‘Very feasible’), n (%)	31 (86.1%)	24 (68.6%)	.077
Agree: “On busy days, there is no time to train women”, n (%)	16 (40.0%)	19 (47.5%)	.499
Assessment that overall workload is reasonable, n (%)	33 (82.5%)	29 (72.5%)	.284

Bolded, italicized results are significant at *p* <.05

Clinic providers reported training slightly more clients in the previous month (average of 16 vs 12) and estimated a slightly higher number of clients that self-injected at the end of training (50% vs. 45% for CHWs). Among providers who had trained clients, a higher percentage of CHWs were ‘Very confident’ in their ability to train women (90% vs. 75%). Despite CHWs taking about 10 min longer for each training as compared with clinic providers (average of 34 min vs. 24 min), a higher percent report that the training time is ‘very feasible’, given their workload (86% vs. 69% for clinic providers). Similarly, a higher percentage of CHWs reported that their overall workload (family planning as well as any other work responsibilities) is reasonable (83% vs. 69% of clinic providers).

### Provider receptivity to self-injection

**Fig. 1:**
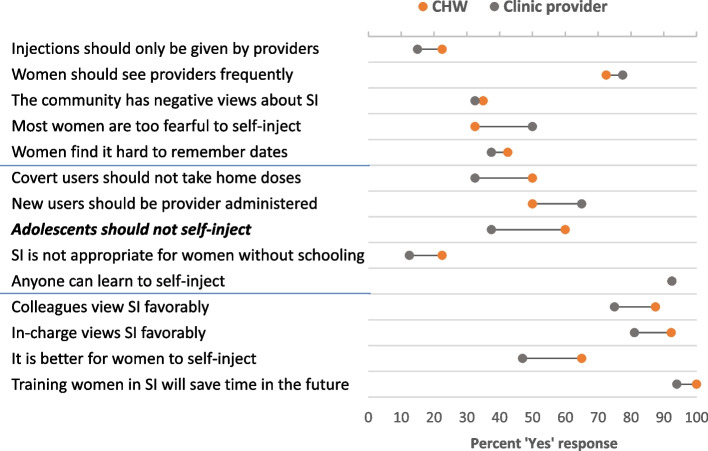
Receptivity towards self-injection among CHWs and clinic providers. Bolded, italicized results are significant at *p* <.05

With respect to their receptivity to self-injection, Fig. 1 shows the percentage of providers who agreed with each statement about self-injection and any limits that should be placed on the type of client who can practice it. The top panel (five items) displays statements expressing general reservations about self-injection. For these general statements, we see few distinctions between clinic providers and CHWs, with the sole exception that a smaller share of CHWs think women are too fearful (33% vs. 50% of clinic providers), though the difference does not reach significance (*p* = .112). For most of these general measures, the majority of providers disagree with the reservation, although most believe that women should visit providers frequently, since ‘providers are the experts.’

The second panel (five items) reflects statements expressing views on who should be permitted to self-inject. A larger share of CHWs than clinic providers express reservations about covert users (50% vs. 33%) and adolescents (60% vs. 38%), with the difference reaching statistical significance for the adolescent measure (*p* = .044). With respect to new users, fewer CHWs agree that women should start with provider-administered injections, as compared with clinic providers (33% vs. 50%). More generally, a substantial share of both provider cadres would place restrictions on who can self-inject, limiting access for covert users (41%), adolescents (49%), and/or new users (58%). A small minority of both types feel that women without education should not self-inject (23% of CHWs vs. 13% of clinic providers), with over 90% of both groups believing that anyone can learn self-injection.

The last panel (four items) are statements generally supportive of self-injection. Here again, CHW attitudes resemble those of clinic providers, but with a somewhat higher percentage of CHWs opining that self-injection is ‘better’ for women (65% vs. 47%).

Calculated as mean self-injection receptivity score across all measures, CHWs and clinic providers show the same level of overall receptivity toward self-injection, both scoring 3.5 on the five-point scale, with higher values representing greater receptivity (not shown).

**Fig. 2:**
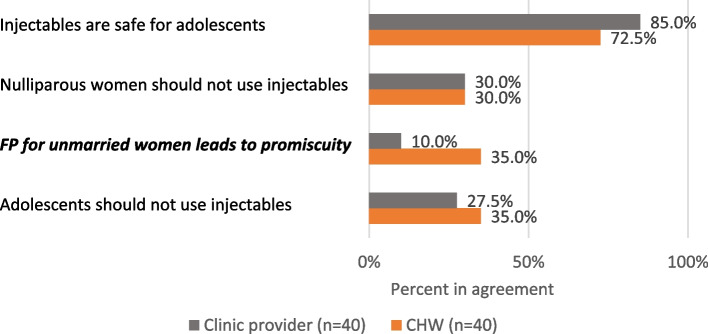
Perspectives on adolescent use of family planning and injectable contraception. Bolded, italicized results are significant at *p* <.05

Taking a closer look at receptivity toward serving adolescent clients (Fig. 2), we see reservations among both cadres, particularly focused on injectable use by adolescents and concern around future fertility. Notably, over one third of CHWs believe that use of family planning will cause unmarried women to behave promiscuously, a significantly greater share than clinic providers (*p* = .007). Half of providers indicated that they had previously received training in adolescent responsive contraceptive services (ARCS), including 58% of CHWs and 43% of clinic providers (not shown). There is no difference in receptivity to adolescents as family planning clients between providers who have received ARCS training and those who have not.

### Client background characteristics

Beginning with their background characteristics, relative to clients seeking services as facilities, clients of CHWs were, on average, slightly older, with higher parity, and were more likely to have received DMPA-SC at their visit (Table 3). With respect to marital status, education, first time method use, and partner support for family planning use, there were no significant differences between clients of CHWs and those visiting facilities. (Because the share of clients self-injecting reflects the sampling approach, which recruited at least one SI client per facility or CHW, the percentage self-injecting should not be interpreted to represent the distribution of self-injectors for participating facilities and CHWs.)

**Table 3:** Characteristics of clients surveyed, both clinic and CHW (client data)

	**Community client (*n* = 120)**	**Clinic client (*n* = 120)**	***P* value**
Age, mean (SD)	***29.8 (0.55)***	***27.4 (0.61)***	.004
Parity, mean (SD)	***3.7 (0.17)***	***3.1 (0.19)***	.015
Married or cohabiting, n (%)	102 (85.0%)	103 (85.8%)	.822
Education n (%)			.614
None	6 (5%)	4 (3.3%)	
Primary	83 (69.2%)	82 (68.3%)	
Secondary O	28 (23.3%)	32 (26.7%)	
Secondary A/University	3 (2.5%)	2 (1.7%)	
Contraceptive experience: New users, n (%)	15 (12.5%)	20 (16.7%)	.360
Partner supports method use, n (%)	107 (89.2%)	103 (85.8%)	.435
Injectable method received at visit, n (%)			.015
DMPA-IM	***20 (16.7%)***	***36 (30.0%)***	
DMPA-SC	***100 (83.3%)***	***84 (70.0%)***	
SC Administration, n (%)	*n* = 100	*n* = 84	.977
Self-injected	26 (26.0%)	22 (26.2%)	
Provider-injected	74 (74.0%)	62 (73.8%)	

Bolded, italicized results are significant at *p* <.05

### Client interest in self-injection

**Fig. 3:**
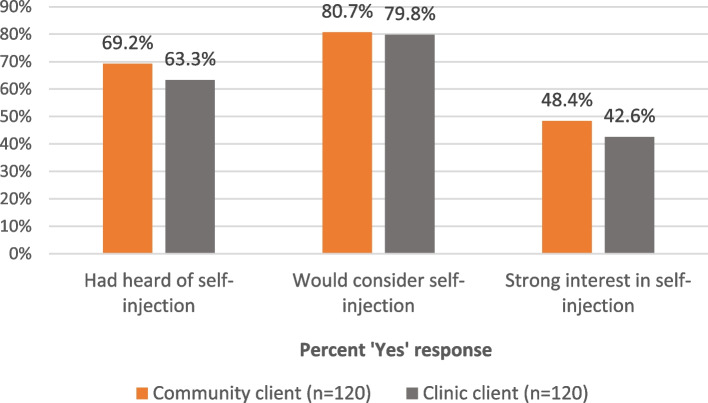
Awareness and interest in SI among injectable clients

As shown in Fig. 3, awareness of and interest in self-injection is relatively high. Roughly two-thirds of these injectable clients had heard of self-injection; four out of five of those not currently self-injecting would consider it (the PMA question) while slightly fewer than half *of those not self-injecting* expressed strong interest (“I am very interested in trying self-injection.”) The level of interest in SI is comparable among clients of CHWs and of facility-based providers. It is interesting to note that over one third of clients who received DMPA-IM (38%) expressed strong interest in SI (not shown).

### Client experiences with self-injection training

**Fig. 4:**
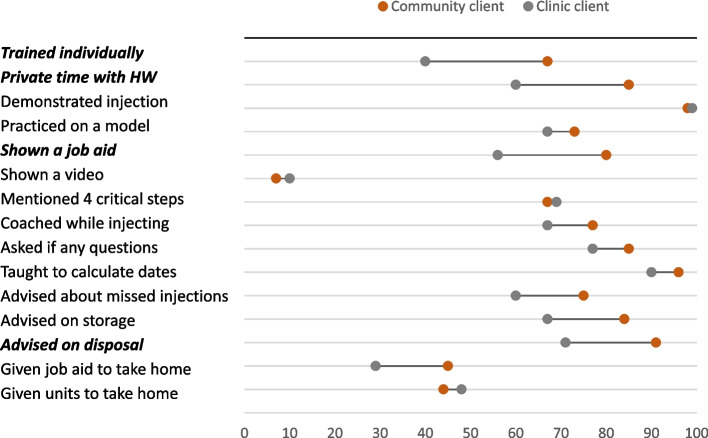
Quality of SI training provided by CHWs and clinic providers. Bolded, italicized results are significant at *p* <.05

Stakeholders are often reticent to permit CHWs to train clients in self-injection out of concern over insufficient capacity or competence among lay health workers. A side-by-side comparison of SI training practices, as reported by clients, suggests their concerns may be misplaced (Fig. 4). Across most measures of quality SI training, CHWs in Uganda equaled or outperformed facility providers. CHWs were significantly more likely to conduct individual training (*p* = .005), ensure private time with the client if trained in a group (*p* = .004), show the client a job aid (*p* = .009), and advise on disposal (*p* = .009). The small sample size precludes detecting statistically significant differences for the other measures with a notable gap, such as: advising what to do if the injection was missed (*p* = .125), advising on storage (*p* = .060), and giving the client a job aid to take home (*p* = .089). Among clients who self-injected at the end of their training, 96% of clients trained by CHWs strongly agreed with the statement: ‘I felt confident that I could self-inject on my own at the end of the training’ (vs. 81% of the clients trained by clinic providers), difference not significant (not shown).

### Client experiences with family planning counseling

**Fig. 5:**
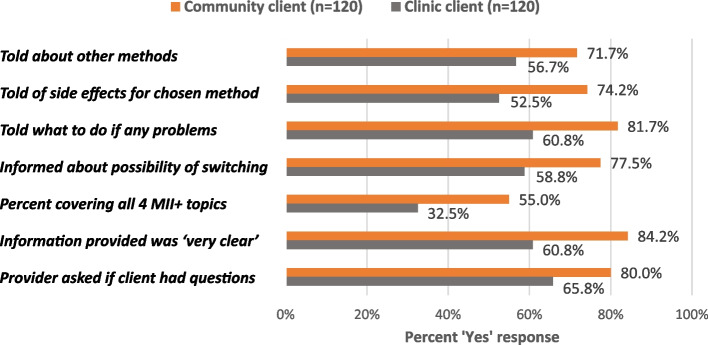
FP counseling content and information quality (MII + and information quality measures). Bolded, italicized results are significant at *p* <.05

SI training is but one aspect of family planning service delivery. The overall quality of family planning counseling is of equal importance given risks that informed choice principles, essential to right-based family planning, are compromised in the context of new method or practice introduction [20–22]. Figure 5 shows the MII + individual and composite measures, as well as indicators for information clarity and solicitation of client questions, as reported by CHW and facility clients. Again, CHWs significantly outperformed their clinic-based colleagues across all measures:—information on other methods (*p* = .015), side effects (*p* = .000), management of side effects (*p* = .000), possibility of switching (*p* = .002), MII + index (*p* = .000), information clarity (*p* = .000) and provider solicited questions (*p* = .014) The finding that only one-third of clinic-based providers covered all four elements of the MII + is cause for concern.

**Fig. 6:**
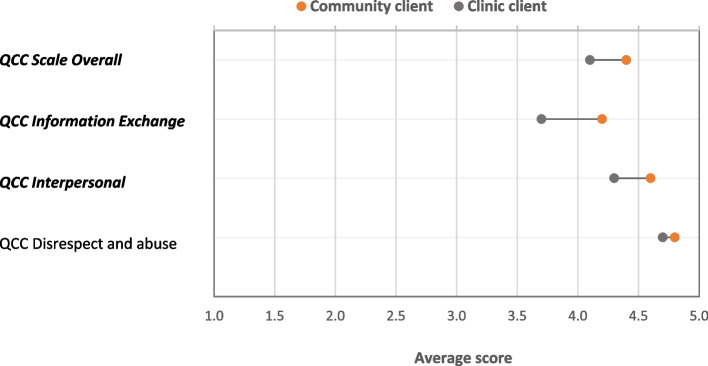
Quality of overall family planning counseling (QCCS, as reported by clients). Bolded, italicized results are significant at *p* <.05

Findings related to the Quality of Contraceptive Counseling scale (QCCS) echo those for counseling content and information quality. As shown in Fig. 6, CHWs were rated significantly higher by their clients for two out of three of the subscales for the QCCS – Information Exchange (*p* = .000) and Interpersonal Relations (*p* = .001) – in addition to the overall scale (*p* = .000). There was no perceived difference between CHWs and clinic providers for the third subscale, Disrespect and Abuse (for which a high score indicates lower Disrespect/Abuse).

Beyond the scales and indices, a simple metric for overall satisfaction with family planning services received during the visit revealed a significantly higher percentage of CHW clients were ‘Very satisfied’ with their visit (77% vs. 65% of facility clients, *p* = .047).

## Discussion

The findings from this study are entirely consistent with the favorable assessment of contraceptive injectable administration by CHWs in sub–Saharan Africa found in the systematic review [12]; indeed, if anything, CHWs in Uganda outperformed their clinic-based colleagues. In particular, the high quality of family planning counseling provided by CHWs in this study – whether measured by the MII + or by the QCCS – is promising. While results are positive for CHWs, the findings with respect to counseling quality among clinic providers, with only one-third covering all four information elements of the MII +, should give program managers pause, suggesting an urgent need for supportive supervision, refresher training, and/or mentorship to reinforce counseling quality.

In the Ugandan context, interest in self-injection, which ranged from four in five injectable users (applying the PMA metric of whether women ‘would consider using’) to just under half (measured as ‘strong interest in SI’) is substantially greater than that found by Wood and colleagues in other SSA settings [3]. Differences may reflect Uganda’s more advanced SI program, coupled with a focus in this study on districts where SI services have been rolled out. National (or even regional) level surveys will necessarily show low awareness and interest if a substantial share of providers, and by extension their clients, are unfamiliar with the concept and do not see self-injection as a normative behavior. More to the point, interest in self-injection was equally high among women served by CHWs relative to women served by clinics. This study suggests that self-care is of interest to women who live far from health facilities, despite access to lay health workers in nearby communities who could administer the injection.

For SI service delivery specifically, training by CHWs tended to be more comprehensive, private, and time-intensive than the training provided at the clinic, and nearly all CHW clients who self-injected had high confidence that they could self-inject independently going forward. As has been found in other studies, CHWs were better able to accommodate the additional time required to train women for self-injection [9]. For clinic providers, the fact that only half of trained clients proceeded to self-inject after being trained may create the perception that women are too fearful to self-inject, contributing to the sense that SI training is not a good use of valuable time. While CHWs had a similar proportion of trained clients self-inject, their opportunity costs to offer SI training, repeatedly as needed, may not create the same disincentive to conduct training.

The modest proportion of trained clients who self-injected in this study among both cadres of providers echoes that found in other studies [8, 23]. In particular, the study by Nai and colleagues in Ghana suggested that adoption of self-injection is a gradual process that, for some women, requires repeated exposure to training [24]. Regardless, relatively low uptake of SI compared with provider-administration has prompted renewed efforts to address how self-injection is messaged to family planning clients. The premise is that, if providers clearly explain the benefits of self-injection and address client fears of pain, uptake will increase. Indeed, Burke’s study in Malawi found higher SI uptake at facilities where providers explained not only the benefits of self-injection but also that SI is safe, easy, less painful (due to the smaller needle), and practiced by other women [25]. However, increases in uptake were not sustained, undermined by supply challenges that led to restrictions on extra units given out for home use, the key rationale for adopting self-injection. Burke’s findings suggest a more complex scenario than the simple explanation that low uptake reflects low demand.

How the concept of self-injection is introduced to women is vitally important, but equally critical is the quality of self-injection training. Our prior research on non-adopters in Uganda found that use of a job aid, demonstrating the injection, allowing women to practice on a model, and comprehensive training content were significant predictors of SI adoption in a multivariate analysis (along with being single and having a supportive partner, the only significant client characteristics identified) [8]. Qualitative work by Porter and colleagues found complementary results in Malawi, with clients who opted not to self-inject citing fear and lack of confidence, even as they observed that their training was of poor quality. Conversely, those who self-injected credited their comprehensive training [22]. The authors conclude their paper with a call for support to providers to overcome time and resource constraints so they can focus on the provision of quality SI training. Their call, coupled with the findings from the current study, should renew our focus on task sharing for SI service delivery and encourage policy approval for CHWs to both administer injections and offer SI training to clients, along with dedicated resources for the training of CHWs in how to counsel women for SI.

This study found a substantial number of providers who had never received training in how to counsel clients for self-injection in districts where SI training had been comprehensively rolled out, and lack of training was an oft-cited reason for failure to offer self-injection services to clients. We also found some reticence to serve certain types of clients. Providers of both cadres would benefit from supportive supervision for SI service delivery, which CHWs in this study were significantly less likely to have received. Supportive supervision coupled with on-the-job training will both catch providers who have not received any training in how to counsel women for SI and address sentiments that may reduce access to SI for specific groups, such as covert users, new users, and adolescents.

With respect to adolescent-responsive contraceptive services specifically, studies have found that traditional training approaches do not have a strong track record for reducing bias against adolescent clients in family planning service delivery. In the current study, the lack of any measurable difference in receptivity to serving adolescents between those who had received ARCS training and those who had not reinforces this assessment. To be effective, a whole systems approach is recommended, with training that includes values clarification and is reinforced through supportive supervision and mentorship [26, 27].

The promise of effective, high quality family planning services at the community level is best realized when countries adopt measures to strengthen the system that supports CHWs. Specifically, the WHO guidelines for CHW programs call for supportive supervision, data collection and feedback to monitor performance and measure the CHW contribution to the family planning program, and integration of CHWs into the supply chain system (among other recommendations) [10]. With respect to the latter, as a recent review of the literature has shown, CHWs are more vulnerable to supply chain disruptions than the facilities with which they are affiliated, and the risk of stock out of essential medicines among CHWs has increased over time [28]. As evident in this study which identified DMPA-SC stock outs as a reason not to offer SI services, a weak supply chain system negatively impacts self-injection, more so than provider-administration of DMPA-SC. When the commodity is in short supply, the ability to take home additional units for self-care – the very rationale for self-injecting – is undermined. When client job aids that instill confidence that women can self-inject effectively are unavailable, adoption of self-injection is likely to wane.

### Study limitations

The small size of this study coupled with the purposive, non-representative sampling approach limits the generalizability of the findings. In particular, the small study size may have resulted in a positively biased sample of CHWs, since only those who counselled injectable clients within the previous two weeks were eligible. The impact of this may be the selection of CHWs who are more active in family planning service delivery, and therefore, may not represent the “typical” CHW. That said, those CHWs who are most active, by definition, are seeing more clients and providing more methods, and therefore, have a disproportionate impact on FP service delivery in communities. Subsequent work should focus on extending and replicating these findings in different geographies and with a wider scope.

Consistent with research ethics principles, the study team engaged the CHWs to identify and recruit potential participants from among their clients (rather than contact clients independently, which would be a privacy violation.) Consequently, we cannot eliminate the possibility that CHWs may have held back the names of clients who they felt would not report favorably. That said, referred clients were required to have been seen in the past two weeks, which greatly limited the pool of clients potentially eligible, and second, clients were randomly selected from a list comprised of clients for all CHWs affiliated with a given facility. As is the case with all evaluations that rely on survey methods, social desirability bias may impact the findings. While client assessments of their counseling experience may be positively biased (inflated), the focus of this paper is on the *difference* in clients’ assessments of clinic- and community-based providers.

## Conclusion

The WHO anticipates a shortfall of 18 million health care workers by 2030, reflecting a pressing need to rapidly advance task sharing and self-care to fill the human resource gap [1]. The findings from this study should reassure stakeholders that, when provided with appropriate, competency-based training and supervision, CHWs are capable of filling that need, reinforcing family planning service delivery while reaching women who face profound geographic access challenges. Moreover, CHWs are better positioned to accommodate in their work schedule the time required for high quality family planning counseling and self-injection training. The vital role of CHWs in bridging the gap between health facilities and households has been evident for decades. The positive results on how and in what fashion self-injection services can be delivered by CHWs reinforce their significant contribution. Optimizing CHWs’ impact in advancing reproductive self-care requires a commitment to providing them with tailored, competency-based training and ongoing supportive supervision. By empowering CHWs with the necessary resources and support, we not only enhance their effectiveness but strengthen the health care systems, ultimately leading to improved access to reproductive health care for all.

## Data Availability

Data from this study is available without restriction from Harvard Dataverse, https://dataverse.harvard.edu/ with the search term “DMPA-SC”.
